# Pentachlorophenol-induced hemotoxicity diminishes antioxidant potential and oxidizes proteins, thiols, and lipids in rat blood: An in vivo study

**DOI:** 10.1016/j.heliyon.2023.e16240

**Published:** 2023-05-13

**Authors:** Nikhil Maheshwari, Aijaz Ahmed Khan, Riaz Mahmood, Samreen Salam

**Affiliations:** a Department of Biochemistry, Faculty of Life Sciences, Aligarh Muslim University, Aligarh 202002, U.P., India; b Department of Anatomy, J. N. Medical College, Aligarh Muslim University, Aligarh 202002, U.P., India

**Keywords:** Pentachlorophenol, Oxidative stress, RBC, Antioxidant status, Liver, Histopathology

## Abstract

Pentachlorophenol (PCP) is an excessively used wood preservative and pesticide, which has resulted in human exposure raising concerns about its potential toxic effects. This study is designed to evaluate the hemotoxicity of PCP in adult rats. Wistar rats were orally administered PCP (25–150 mg/kg bw) for five days while untreated (control) rats received corn oil. Animals were sacrificed, blood was taken and fractionated into plasma and red blood cells (RBC). PCP administration increased methemoglobin formation but decreased methemoglobin reductase activity. Significantly increased hydrogen peroxide level indicates initiation of oxidative stress condition in blood. PCP increased the oxidation of thiols, proteins and lipids, lowered glutathione levels, and compromised the antioxidant status of RBC in treated rats. Enzymes of the pathways of glucose breakdown, glycolysis and phosphogluconate pathway, were inhibited. Markers of liver damage were increased in the plasma of PCP-treated rats suggesting hepatotoxicity. This was confirmed by histopathological analysis of stained liver sections. Activity of xanthine oxidase, a reactive oxygen species (ROS) generating pro-oxidant enzyme, was increased. These hematological changes could be a result of the increased generation of ROS or direct chemical transformation by transient reaction species. These results show that PCP induces redox imbalance, diminishes antioxidant potential, inhibits metabolic pathways, and oxidizes cellular components in rat blood.

This study suggests an elaborated possible molecular mechanism of PCP toxicity, and similar compounds so that methods can be devised to minimize its damaging effect.

## Highlights

• Pentachlorophenol (PCP) increases pro-oxidant enzyme xanthine oxidase in plasma.
• PCP enhances oxidation of thiols, proteins and lipids in rat blood.
• PCP impairs the antioxidant status and inhibits enzymes of glucose breakdown.
• PCP increases plasma AST and ALT levels and causes histological abrasions in liver.
• A leading step to device curative methods against PCP-induced hemotoxicity.

## 1 Introduction

Chlorophenols are intermediate compounds used in the manufacture of agrochemicals, biocides, dyes, and pharmaceuticals. PCP is a stable, persistent and highly toxic chlorophenol that is primarily used in wood preservation. PCP is commercially used to manufacture pulp and paper, paints, gaur gums and to control molds in petroleum drilling. It is used in defoliant, pre-harvest desiccant, and as an anti-termite agent. PCP is a major component of several fungicides, molluscicides, rodenticides, insecticides, and biocide products [1]. Commercial formulations like Dowicide-5, Penwar 1–5 (EPA Reg. No.7234-7), and Pentacon-10 (EPA Reg. No. 61483-59) contain very high amounts of PCP (85%, 25.6%, and 8.96%, respectively) [2]. Salts of PCP at 12.5% in dry formulations and 2.5–35.7 g/L in liquid (US Patent No. 3846114A) were used to combat the moss “blue stain” in pine timber and eliminate the decaying of wood in the ground by *Lentinus lepideus* [3].

The global production of PCP in 1981 was evaluated to be 90,000 tons/year. In 2003, 3010 tons of Na-PCP was consumed in China, 60% of which (1806 tons) was used to control schistosomiasis. China still produces 5000 tons/year of PCP as reported in 2010 [4,5]. In 2010, the USA produced 7256 tons/year of PCP and is still manufacturing PCP at 3 facilities located in Alabama and Kansas, USA, and Mexico [4]. India was the leading exporter of Na-PCP and manufactured 1800 tons/year in the states of Maharashtra and West Bengal. In 2016 several industries were registered to manufacture PCP in India, China, Japan, Israel, Canada, South Africa and Netherlands (1 industry), United Kingdom (3 industries), Mexico, Germany, and Switzerland (2 industries), USA (10 industries) [1]. Latin-North-America, Europe, Asia Pacific, Middle East and Africa are the global markets of zinc salt (zinc bis(pentachlorophenolate) and sodium salt (sodium pentachlorophenate) of PCP [6,7]. The United States-Environmental Protection Agency (US-EPA) also lifted the ban on the commercial trade of wood preservatives containing PCP till 2027 [8].

The US-EPA set a maximum contamination level (MCL) for PCP in drinking water as 1 μg/L. The maximum concentration allowed in drinking water (bottled or tap water) is 3.5 nM [9] and 34 nM according to the provisional guidelines of WHO [10]. PCP in μmolar concentrations has been reported in groundwater whilst remarkably higher levels, 0.5–0.7 mM PCP, have been found in industrial effluents and waste [11]. Humans are exposed to PCP through drinking water disinfected with chlorinated oxidants and water resources polluted with PCP [12]. Exposure to PCP also occurs through the indoor air of treated log homes and industries that supply wood, dipped and brushed with PCP [13]. When humans are exposed to PCP through ingestion and inhalation, it damages the kidney [14], intestine [15], cardiovascular system and liver (jaundice) [16]. Workers at lumber mills and wood treatment facilities can inhale about 10.5–154 mg PCP/day and absorb about 35 mg PCP/day via the skin [17]. Blood PCP concentrations were highest in workers directly involved in PCP-utilizing commercial units and also those residing in PCP-treated log homes [18]. In chronically exposed workers, PCP concentrations of 14.0 mg/L in blood and 8.4 mg/L in serum were reported [1].

Chronic human oral exposure to PCP results in acute upper respiratory tract inflammation, bronchitis, hemotoxicity [19], aplastic anemia [20], red cell aplasia [20], Hodgkin's disease, acute leukemia and adenomas in the liver and kidney [16,17]. Chronic PCP toxicity lowers RBC count and hemoglobin level leading to anemia [21]. Clinical and experimental reports document the hematologic, carcinogenic [22], and mutagenic [23] effects of PCP and its chemical contaminants [20]. International Agency for Research and Cancer (IARC) 2019 categorized PCP as a Group 2B possible human carcinogen. Since PCP poisoning has no antidote [24], several (1495) incidents of accidental and intentional (suicidal) PCP poisoning [25], including some fatal cases, have been reported [1,13,26].

Blood and RBC represent a major and important target of PCP that enters the body through any route. PCP is rapidly absorbed by the intestine upon oral intake, with 100% bioavailability, and its blood level peaks in a few hours. PCP is then distributed to other vital organs by circulating blood. In this study, we report that PCP elevates ROS formation that oxidizes lipids, proteins and thiols, compromises antioxidant (AO) defenses, and oxidatively damages the RBC membrane.

## 2 Materials and methods

Chemicals: PCP (97%, purity) was obtained from Sigma-Aldrich, USA. All other chemicals used were of analytical grade from Sigma-Aldrich (USA), Himedia (Mumbai, India) or Sisco Research Laboratory (Mumbai, India).

Animals: Adult, male Wistar rats of about 150 g were bought from the National Institute of Biologicals (CPCSEA Reg. No. 824/GO/RBiBT/S/04/CPCSEA), Ministry of Health and Family Welfare, Government of India, India.

Diet: Rats were given a standard diet purchased from Aashirwad Industries, Chandigarh, India.

### 2.1 Treatment of animals

Rats were first acclimatized for 7 days on a standard diet and water *ad libitum* and then divided into five groups (control and 4 PCP treated), with 5 animals in each group. PCP was solubilized in corn oil and orally administered to rats at doses of 25, 50, 100, and 150 mg/kg bw once a day for 5 days. These doses were below 211 mg/kg bw, the reported oral LD_50_ value of PCP in rats [27]. Rats in the control group received only corn oil by gavages.

Rats were killed 24 h after giving the last dose of PCP, under chloroform anesthesia. Chloroform inhalation is a widely used method of rodent euthanasia. After opening the peritoneum cavity, blood was collected through cardiac puncture in heparin-coated tubes. Blood was spun at 1200 rpm for 12–15 min, the plasma (supernatant) was collected, aliquotted and used immediately or frozen at −80 °C for later analysis. The RBC in pellet were washed 3 times with phosphate-buffered saline (PBS; 0.9% sodium chloride in 0.01 M sodium phosphate buffer, pH 7.4). Washed RBC were then lysed by adding ten volumes of 5 mM sodium phosphate buffer, pH 7.4, kept at 4 °C for 2 h, and spun at 3000 rpm for 8–10 min to remove any cell debris. The supernatants (cell lysates) were used at once or kept at −80 °C.

Hemoglobin (Hb) content in RBC lysates was quantified using Drabkin's reagent obtained from Coral Clinical Systems, Goa, India [28]. Concentration of proteins in plasma was quantified as described by Lowry et al. [29]. A standard plot of different concentrations of bovine serum albumin (BSA) was prepared to determine the protein content.

### 2.2 Hemoglobin oxidation and markers of oxidative stress

The methemoglobin (MetHb) level in RBC lysates was quantified from the absorbance of cell lysates at 560, 576, and 630 nm wavelengths after suitable dilution [30]. MetHb reductase activity in RBC lysates was monitored by following the NADH-dependent reduction of 2,6-dichlorophenolindophenol at 600 nm [31].

The major non-enzymatic antioxidant (AO) glutathione (GSH), total sulfhydryl (T-SH), carbonyl content, lipid peroxidation (LPO) and H_2_O_2_ levels were measured in both RBC lysates and plasma. The GSH concentration was determined in protein-free samples as explained by Beutler [32]. Proteins were precipitated using 1.66% metaphosphoric acid and pelleted by centrifuging samples at 12,000 rpm for 10 min. After adding 5,5′-dithiobis-2-nitrobenzoic acid (DTNB) to the supernatants, the absorbance of yellow 5-thionitrobenzoate anion formed was read at 412 nm. Protein carbonyls, a measure of protein oxidation, yield hydrazone adducts with 2,4-dinitrophenylhydrazine which absorb at 360 nm [33]. T-SH groups were quantified by DTNB method as described by Sedlak and Lindsay [34]. LPO was quantified from the reaction of its marker malondialdehyde with thiobarbituric acid to yield a pinkish-red complex that absorbs at 535 nm [35]. H_2_O_2_ level was measured by using ferric-xylenol orange coloring reagent, which forms a blue-purple complex in an acidic medium; 100 mM sorbitol acts as a color enhancer in the reaction [36]. Nitric oxide (NO) level was determined using Greiss reagent [0.1% N-(1-naphthyl)ethylenediamine dihydrochloride, 1% sulphanilamide] as explained by Miranda et al. [37].

### 2.3 Antioxidant power

Antioxidant power (AO) power was determined in RBC lysates and plasma by the free radical quenching (DDP^•^ to DPPH) and metal-reducing (Fe^3+^, Cu^2+^, Mo^6+^) ability of AOs in samples. In the FRAP (ferric reducing antioxidant power) assay, Fe^3+^ is reduced to Fe^2+^ by sample AOs and then Fe^2+^ reacts with 2,4,6-tris(2-pyridyl)-*s*-triazine to yield a blue Fe^2+^-tripyridyltriazine complex that absorbs at 593 nm [38]. In CUPRAC (cupric reducing antioxidant capacity) method, Cu^2+^ ions are reduced to Cu^+^ and Cu^+^ gives a colored complex with neocuproine [39]. AOs in the samples convert Mo^6+^ to Mo^5+^ in phosphomolybdenum assay, and a green-colored phosphate-Mo^5+^ complex is produced [40]. DPPH (2,2-diphenyl-1-picrylhydrazyl) method was employed to monitor the free radical quenching power of cells. The purple-colored DDP^•^ free radical in the aqueous solution accepts a hydrogen atom from AOs in samples which quenches DDP^•^ back to pale yellow DPPH. In DPPH assay, hemolysate/plasma (0.02 mL) was mixed with 10 mM sodium phosphate buffer pH, 7.4 (0.48 mL), in duplicate sets. Then, 0.5 mL of 0.1 mM DPPH (in methanol) was added in one set while 0.5 mL of methanol was added in the other set. After incubating the reaction mixture in the dark for 30 min at room temperature, it was centrifuged at 11,000 rpm for 12 min and absorbance of supernatants was noted at 517 nm against a reference containing 0.5 mL sodium phosphate buffer and 0.5 mL DPPH solution [41].

$$DPPH\;\left(\%\;quenching\right)=\frac{OD{\;}_{Reference}\;–\;OD{\;}_{Sample}}{OD\;Reference}\times 100$$

### 2.4 Antioxidant enzymes

The specific activities of catalase, superoxide dismutase (SOD), glutathione peroxidase (GPx) and thioredoxin reductase (TR) were determined in plasma and RBC lysates. Catalase activity was measured from the conversion of H_2_O_2_ to water and O_2_ which decreases the sample absorbance at 240 nm [42]. SOD activity was followed by inhibition of the auto-oxidation of pyrogallol [43]. TR activity was monitored using DTNB which is enzymatically cleaved, in the presence of reduced nicotinamide adenine dinucleotide phosphate (NADPH), to give the yellow 5-thionitrobenzoate anion. The enhanced absorbance at 412 nm was noted [44]. GPx activity was monitored by following the decrease in absorbance at 340 nm upon oxidation of NADPH to oxidized nicotinamide adenine dinucleotide phosphate (NADP^+^) in presence of GSH [45]. Glutathione reductase (GR) and glutathione S-transferase (GST) activities were determined in RBC lysates only. GR was assayed from the oxidation of NADPH to NADP^+^ along with enzymatic conversion of GSSG to GSH and the decrease in absorbance was recorded at 340 nm [46]. GST activity was determined using 1-chloro-2,4-dinitrobenzene and GSH as substrates [47]. Xanthine oxidase (XO) was assayed in plasma by the method of Shintani [48]. The increase in absorbance at 290 nm was noted upon enzymatic cleavage of xanthine to uric acid. A molar extinction coefficient of 1.22 × 10^4^ M^−1^cm^−1^ was used to calculate the enzyme activity.

### 2.5 Membrane-bound enzymes

Acetylthiocholinesterase (AChE), total and Na^+^K^+^ATPase were assayed in RBC lysates. AChE hydrolyzes S-acetylthiocholine iodide to thiocholine; the latter dissociates the disulfide bond of DTNB producing yellow 5-thionitrobenzoate anion that absorbs at 412 nm [49]. The activity of total and Na^+^K^+^ATPase were determined by measuring the inorganic phosphate (Pi) liberated upon ATP cleavage in the absence and presence of 1 mM ouabain. The Pi yields a blue complex upon reaction with Taussky and Shorr reagent [50].

### 2.6 Metabolic enzymes

Hexokinase (HK) activity was determined by the method of Bergmeyer et al. [51]. The method involves the reduction of NADP^+^ to NADPH in a coupled enzymatic reaction catalyzed by HK and glucose-6-phosphate dehydrogenase (G6PD). Pyruvate kinase (PK) converts phosphoenolpyruvate to pyruvate along with ATP hydrolysis. Lactate dehydrogenase (LDH) further converts pyruvate to lactate. This is accompanied by oxidation of NADH to NAD^+^ leading to a decrease in the absorbance of solution at 340 nm [52]. G6PD was assayed from the reduction of NADP^+^ to NADPH in presence of glucose 6-phosphate. Resulting increase in absorbance at 340 nm was recorded [53]. In the LDH assay, the decrease in absorbance was followed at 340 nm upon conversion of NADH to NAD^+^ in the presence of sodium pyruvate [54]. Acid phosphatase (ACP) assay used p-nitrophenyl phosphate as the substrate which generates yellow p-nitrophenol on hydrolysis, causing an increase in sample absorbance at 415 nm [55]. Glyoxalase-I (GLO-I) catalyzes the isomerization of its substrate hemithioacetal to S-lactoylglutathione upon addition of cell lysate containing the enzyme. The change in absorbance at 240 nm was recorded [56].

### 2.7 Plasma parameters

Aspartate aminotransferase (AST) and alanine aminotransferase (ALT) activities were measured in plasma using kits from Span Diagnostics (Surat, India). The concentration of end products formed (oxaloacetate in AST and pyruvate in ALT) was determined spectrophotometrically by their reaction with 2,4-dinitrophenylhydrazine under alkaline conditions. The hydrazone adducts formed absorb at 510 nm [57]. Glucose and inorganic phosphate were determined in protein-free plasma samples. Plasma proteins were precipitated by trichloroacetic acid (5%, final concentration), pelleted by spun at 10,000 rpm for 12 min and the protein-free supernatants were used further. Glucose concentration was determined by the o-toluidine method in protein-free supernatants. Samples (0.2 mL) were mixed with 2 mL o-toluidine reagent (1.5 g thiourea dissolved in a solution containing 60 mL o-toluidine and 940 mL glacial acetic acid) and kept at 100 °C for 12 min. Samples were cooled to room temperature and their absorbance at 630 nm was noted. Glucose concentration was calculated using a calibration curve prepared from known concentrations of glucose [58]. The serum level of inorganic phosphate was quantified using the method of Taussky and Shorr [59].

### 2.8 Liver histopathology

Small pieces of liver were cut and instantly preserved in a 10% formalin solution. Paraffin blocks were prepared after fixing the tissue in wax. Then microscopic sections of 10 μm thickness were cut from paraffin blocks using a microtome. Tissue sections were mounted on glass slides and after hematoxylin and eosin staining the slides were observed at 1000× magnification under a trinocular microscope (Olympus B×40, Japan) [60].

### 2.9 Statistical analysis

All the experiments were performed using blood from five different rats and dispersion of set is reported as standard deviation and shown as error values. The Bartlett's test was applied to check the homogeneity of variance and a P value of 0.861 (0.754–0.961) was found, which is greater than α (0.05). Statistical significance was determined by One-way ANOVA using the software program Origin Pro 8.0 (USA). Results were considered significant when probability value of α was ≤0.05, analyzed by the Tukey post-hoc test.

## 3 Results

In this study, we evaluated the toxicity of PCP in rat blood and detailed biochemical analysis were carried out on plasma and isolated RBC, including markers of oxidative stress and membrane integrity, antioxidant status, and enzymes of glucose metabolism.

### 3.1 Hemoglobin (Hb) oxidation and markers of oxidative stress

**Fig. 1:**
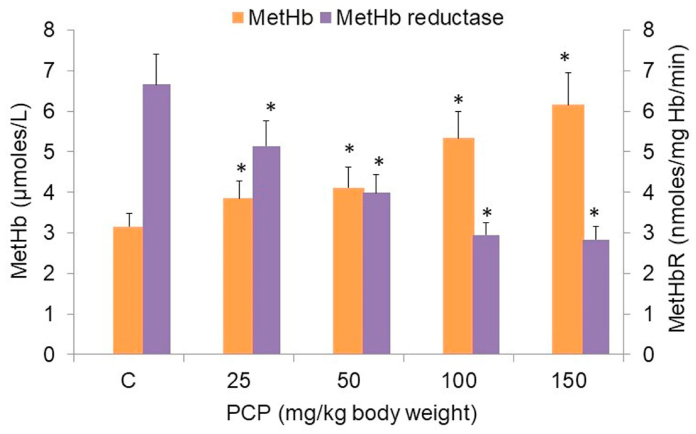
Methemoglobin (MetHb) levels and methemoglobin reductase (MetHbR) activity in RBC lysates. C, control. Data are given as mean ± standard error of 5 different samples. At p ≤ 0.05 results are significantly different (*) from control, analyzed by one-way ANOVA.

**Fig. 2:**
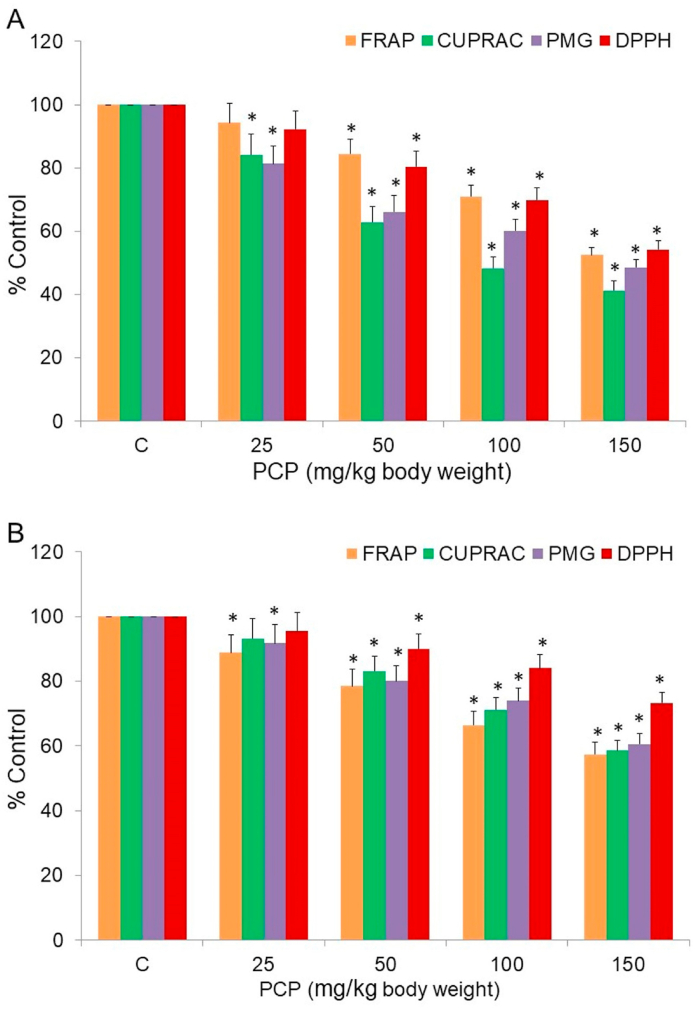
PCP-mediated changes in antioxidant power of (A) RBC lysates and (B) plasma, determined by FRAP, CUPRAC, PMG and DPPH assays and reported in % change from control. Data are given as mean ± standard error of 5 different samples. At p ≤ 0.05 results are significantly different (*) from control, analyzed by one-way ANOVA. DPPH, 2,2-diphenyl-1-picrylhydrazyl; CUPRAC, cupric reducing antioxidant capacity; FRAP, ferric reducing antioxidant power; PMG, phosphomolybdenum green. (For interpretation of the references to color in this figure legend, the reader is referred to the Web version of this article.)

PCP treatment increased MetHb formation due to the conversion of Fe^2+^in Hb to Fe^3+^; in the highest PCP-treated group (150 mg/kg bw) it was twofold of the control values (Fig. 1). MetHb reductase activity was significantly decreased in cell lysates and in the highest PCP-treated group it was only 40% of the control value (Fig. 2). GSH levels in RBC lysates and plasma were lowered to 43% in a PCP dose-dependent manner (Table 1). Total sulfhydryl content was also decreased to 48% in RBC lysates and 70% in plasma (Table 1). PCP administration increased lipid peroxidation to 2.7 fold of control in RBC lysates and 1.7 fold in plasma (Table 1). A 3 fold increase in protein oxidation was noted in RBC lysates and 2 fold in plasma of rats given the highest PCP dose, as compared to control groups (Table 1). PCP treatment increased the production of H_2_O_2_, a non-radical ROS, to 3.5 fold in RBC lysates and 2 fold in plasma. The production of nitric oxide was also increased in blood (Table 1).

**Table 1:** PCP-mediated changes in markers of oxidative stress in RBC lysates and plasma of rat blood.

	PCP (mg/kg body weight)				
	Control	25	50	100	150
**GSH**					
RBC	36.56 ± 4.25	25.48 ± 2.14*	20.53 ± 1.84*	16.39 ± 1.91*	12.19 ± 1.53*
Plasma	6.51 ± 0.81	5.48 ± 0.72	4.75 ± 0.64*	3.82 ± 0.39*	3.13 ± 0.46*
**Total-SH**					
RBC	1.25 ± 0.27	1.03 ± 0.21	0.78 ± 0.17*	0.64 ± 0.093*	0.60 ± 0.081*
Plasma	0.31 ± 0.045	0.30 ± 0.041	0.27 ± 0.033	0.23 ± 0.026*	0.22 ± 0.028*
**PO**					
RBC	11.0 ± 1.83	13.66 ± 1.78	17.43 ± 2.11*	25.59 ± 2.92*	33.78 ± 4.05*
Plasma	11.77 ± 1.37	15.35 ± 1.61*	17.63 ± 2.08*	19.57 ± 1.76*	23.58 ± 2.05*
**LPO**					
RBC	0.89 ± 0.112	1.09 ± 0.164	1.36 ± 0.193*	1.85 ± 0.267*	2.43 ± 0.362*
Plasma	1.29 ± 0.164	1.52 ± 0.197	1.73 ± 0.221*	1.87 ± 0.282*	2.18 ± 0.302*
**H**_**2**_**O**_**2**_					
RBC	3.61 ± 0.451	4.84 ± 0.537*	5.78 ± 0.711*	8.12 ± 0.930*	12.71 ± 1.64*
Plasma	2.07 ± 0.255	2.23 ± 0.286	2.53 ± 0.302*	3.05 ± 0.381*	3.97 ± 0.518*
**NO**					
RBC	0.43 ± 0.056	0.58 ± 0.073*	0.67 ± 0.082*	0.87 ± 0.095*	1.05 ± 0.167*
Plasma	0.36 ± 0.047	0.54 ± 0.068*	0.65 ± 0.073*	0.76 ± 0.096*	0.94 ± 0.11*

GSH, LPO, PO, H_2_O_2_ and NO levels are in nmoles/mg Hb (RBC lysates) or nmoles/mg protein (plasma) and total SH is in μmoles/mg Hb or protein, respectively.

Data are given as mean ± standard error of 5 different samples.

At p ≤ 0.05 results are significantly different (*) from control, analyzed by one-way ANOVA.

GSH, reduced glutathione; SH, sulfhydryl group; PO, protein oxidation; LPO, lipid peroxidation; H_2_O_2_, hydrogen peroxide; NO, nitric oxide.

### 3.2 Antioxidant status

Various assays of free radical quenching and metal ion reduction were used to determine the AO capacity/power of plasma and RBC lysates from control and PCP-treated rats. The gross AO capacity of RBC was reduced to half (Fig. 2A) and decreased by 40% in plasma (Fig. 2B) in 150 mg PCP/kg bw treated rats when compared to control animals.

The effect of oral administration of PCP on antioxidant enzymes was determined next. SOD activity was reduced to 43% in RBC and 53% in plasma (Table 2). The catalase activity in RBC was found to be 48% and 43% in RBC and plasma of the highest PCP-treated rats when compared to control animals. GPx activity was inhibited to 40% in RBC and 43% in plasma of PCP-treated rats (Table 2). TR activity was reduced to 56% in RBC and 40% in plasma of PCP-treated rats while GR activity was also compromised to 55% in RBC of PCP-treated rats (Table 2). Oral administration of PCP enhanced the GST activity in RBC lysates by 1.8 fold when compared to control animals (Table 2). XO activity was enhanced 1.5 fold in plasma of PCP-treated rats (Table 2).

**Table 2:** PCP-induced alterations in the activities of major antioxidant defense enzymes in rat blood.

	PCP (mg/kg body weight)				
	Control	25	50	100	150
**Catalase**					
RBC	25.55 ± 3.03	20.08 ± 2.64*	17.92 ± 2.36*	14.59 ± 1.96*	12.30 ± 1.58*
Plasma	15.35 ± 2.02	13.07 ± 1.88*	10.31 ± 1.59*	8.63 ± 1.01*	6.67 ± 0.87*
**SOD**					
RBC	50.26 ± 6.69	40.89 ± 5.86*	37.07 ± 4.74*	26.90 ± 3.77*	21.83 ± 3.35*
Plasma	31.90 ± 4.97	28.31 ± 3.80	24.85 ± 3.52*	19.63 ± 3.07*	17.54 ± 2.15*
**GPx**					
RBC	40.04 ± 5.67	31.15 ± 4.56*	24.77 ± 3.24*	24.44 ± 3.01*	23.63 ± 2.85*
Plasma	22.81 ± 3.10	19.09 ± 2.64	15.84 ± 2.07*	14.17 ± 1.82*	13.1 ± 1.68*
**TR**					
RBC	138.02 ± 14.57	122.27 ± 11.63	108.3 ± 13.36*	86.45 ± 10.08*	78.0 ± 9.68*
Plasma	80.46 ± 10.55	70.44 ± 9.34*	52.93 ± 8.67*	45.32 ± 6.94*	32.48 ± 5.05*
**GST**					
RBC	21.07 ± 2.11	23.06 ± 2.66	25.63 ± 3.31	28.9 ± 3.90*	38.74 ± 3.59*
**GR**					
RBC	72.96 ± 10.22	61.58 ± 6.12*	57.96 ± 5.26*	51.70 ± 5.60*	40.92 ± 4.08*
**XO**					
Plasma	1.29 ± 0.183	1.43 ± 0.202	1.53 ± 0.231	1.81 ± 0.217*	2.01 ± 0.269*

Specific activity of SOD is in units/mg Hb (RBC lysates) or units/mg protein (plasma). Catalase, GPx and TR are in nmoles/min/mg Hb or protein, GST and GR in nmoles/min/mg Hb and XO in nmoles/min/mg protein.

Data are given as mean ± standard error of 5 different samples.

At p ≤ 0.05 results are significantly different (*) from control, analyzed by one-way ANOVA.

SOD, superoxide dismutase; GPx, glutathione peroxidase; TR, thioredoxin reductase; GST, glutathione S-transferase; GR, glutathione reductase; XO, xanthine oxidase.

### 3.3 Membrane-associated enzymes

**Fig. 3:**
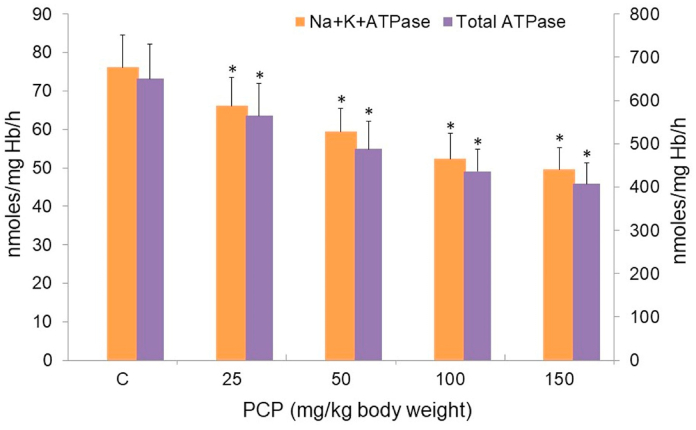
PCP-mediated alterations in the activities of membrane-bound enzymes, total ATPase and Na+K+ATPase. C, control. Data are given as mean ± standard error of 5 different samples. At p ≤ 0.05 results are significantly different (*) from control, analyzed by one-way ANOVA. ATPase, adenosine triphosphatase.

**Fig. 4:**
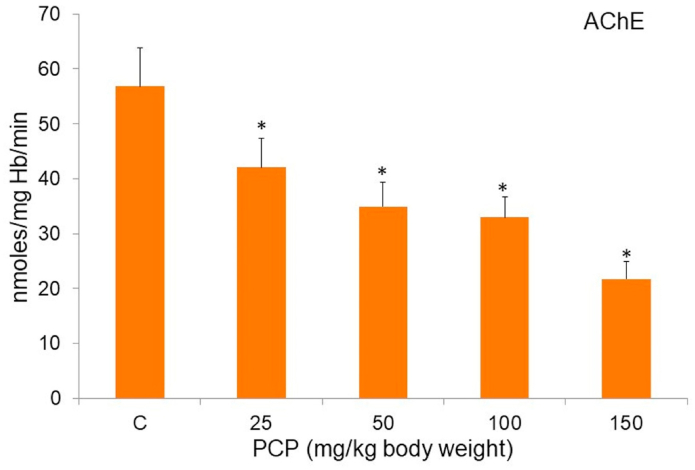
PCP-mediated alterations in the activity of membrane-bound enzyme AChE. C, control. Data are given as mean ± standard error of 5 different samples. At p ≤ 0.05 results are significantly different (*) from control, analyzed by one-way ANOVA. AChE, acetylcholinesterase.

The activities of membrane-associated enzymes, ATPases, and AChE, were determined to check RBC membrane integrity. The activities of both enzymes were inhibited in PCP-treated rats. The activities of total ATPase and Na^+^K^+^ATPase were lowered to 62% and 65% in RBC of PCP-treated rats (Fig. 3). AChE activity was also reduced to 38% in RBC of PCP-treated rats in comparison to control rats (Fig. 4).

### 3.4 Metabolic enzymes

Specific activities of all metabolic enzymes were altered in PCP-treated rats. PCP administration to rats inhibited HK, PK, G6PD and ACP activities to 64%, 56%, 53% and 59% of control rats in a PCP dose-dependent manner, respectively (Table 3). LDH and GLO-1 activities in RBC of PCP-treated rats were increased 2 and 3 fold, respectively, when compared to control animals (Table 3).

**Table 3:** PCP-induced changes in activities of metabolic enzymes in RBC lysates.

	PCP (mg/kg body weight)				
	Control	25	50	100	150
HK	76.6 ± 9.02	64.72 ± 8.23	58.72 ± 7.41*	53.63 ± 6.61*	49.33 ± 5.84*
PK	51.55 ± 6.75	45.65 ± 6.41	38.0 ± 5.13*	32.02 ± 5.01*	29.14 ± 3.36*
LDH	16.81 ± 2.81	19.85 ± 2.03	22.0 ± 2.75*	30.08 ± 3.91*	36.63 ± 5.11*
G6PD	116.4 ± 15.10	106.9 ± 12.62	88.6 ± 10.61*	73.61 ± 9.18*	62.36 ± 8.03*
ACP	25.39 ± 3.03	23.24 ± 2.81	20.47 ± 2.52*	17.65 ± 2.14*	14.72 ± 1.28*
GLO-1	9.94 ± 1.71	14.60 ± 2.01*	17.58 ± 2.35*	21.32 ± 2.58*	28.96 ± 3.59*

Specific activity of all enzymes is in nmoles/min/mg Hb.

Data are given as mean ± standard error of 5 different samples.

At p ≤ 0.05 results are significantly different (*) from control, analyzed by one-way ANOVA.

HK, hexokinase; PK, pyruvate kinase; LDH, lactate dehydrogenase; G6PD, glucose 6-phosphate dehydrogenase, ACP, acid phosphatase; GLO-1, glyoxalase-1.

### 3.5 Plasma markers of oxidative stress

**Fig. 5:**
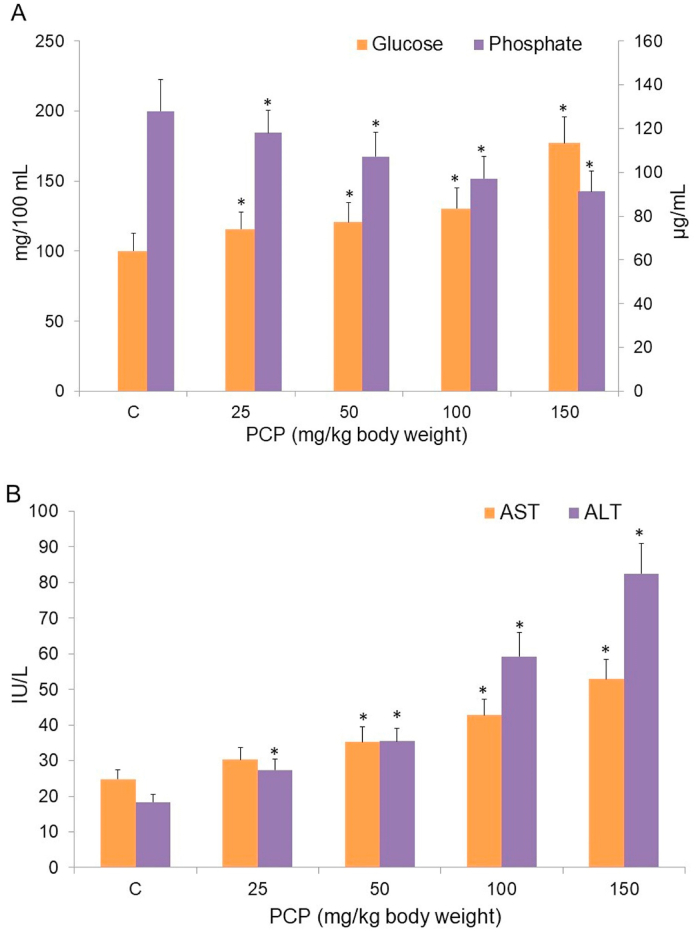
PCP-mediated changes in (A) glucose and inorganic phosphate levels (B) AST and ALT levels, in plasma of rat blood. C, control. Data are given as mean ± standard error of 5 different samples. At p ≤ 0.05 results are significantly different (*) from control, analyzed by one-way ANOVA. AST, aspartate aminotransferase; ALT, alanine aminotransferase.

A significant change in enzymatic and non-enzymatic parameters in plasma of PCP-treated rats was found. The plasma glucose level was increased from 100 mg/dl to 177 mg/dl in treated rats (Fig. 5A). The Pi level in plasma was decreased to 70% in PCP-treated rats when compared to control rats (Fig. 5A). A 2.1 fold increase in AST and 4.5 fold increase in ALT activities was seen in plasma relative to control group (Fig. 5B).

### 3.6 Histopathology of liver

**Fig. 6:**
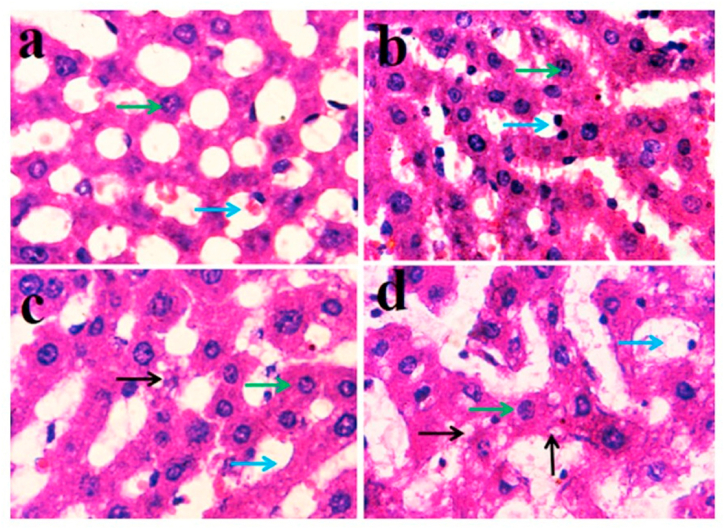
Representative photomicrographs of rat liver from (a) untreated control group, and (b, c and d) treated with PCP at 25, 100 and 150 mg/kg bw, respectively. Green arrows indicate hepatocytes, blue arrows hepatic sinusoids, and black arrows intracytoplasmic vesicles wherever present in the groups. Initial magnification 1000X. H & E stain. (For interpretation of the references to color in this figure legend, the reader is referred to the Web version of this article.)

Histopathological images from the liver of untreated control rats showed discrete morphology of hepatocytes; the sinusoids are not congested and hepatocytes are devoid of vesicles and vacuoles (Fig. 6a). The PCP-treated groups show gradual changes in terms of blurring of boundaries, and the apparent progressive clumping/fusion of hepatocytes, as compared to the control. The intracytoplasmic vesicles are not seen in control and 25 mg/kg bw treated groups (Fig. 6a and b). In 100 mg/kg bw treated group, they are small and infrequent (Fig. 6c) while in 150 mg/kg bw treated group the vesicles are numerous, present in clusters and can be seen to affect majority of the cells in the field (Fig. 6d).

## 4 Discussion

PCP is an inexpensive biocidal agent that has broad applications in wood preservation, industries, and agriculture. PCP concentration of 190 ppm (190 mg/L) is reported in industrial effluents and wastewater resources [1,11]. PCP easily crosses the skin, respiratory tract and gets absorbed in the intestine due to its high lipophilicity [61]. It is then distributed to various vital organs and in the body fluids [62,63]. PCP toxicity likely involves enhanced production of ROS which leads to a condition of oxidative stress [64].

Hb is an abundantly (≥95% of total proteins) found iron-containing oxygen-transport metalloprotein in the RBC of almost all vertebrates. Due to its abundance, free radicals/ROS mainly target Hb in these cells and oxidize the prosthetic group ferroheme to ferriheme thereby converting Hb to MetHb. The oxidation of Fe^2+^ to Fe^3+^ makes Hb inactive for transporting oxygen to tissues [65]. The NADH-dependent enzyme MetHb reductase catalyzes the conversion of MetHb back to Hb. Inhibition of MetHb reductase causes an abnormal increase of MetHb which has high O_2_ binding affinity but does not unload oxygen in the tissues. MetHb is, therefore, inactive as an O_2_ transporter. Increased MetHb levels can be observed during toxic insult, which compromises the RBC's reducing capacity and results in functional anemia. The electron released upon Hb oxidation to MetHb can be taken up by O_2_ to generate superoxide radicals (O_2_^•-^) which are then converted to H_2_O_2_. Free iron reacts with H_2_O_2_ and initiates the Fenton reaction to form the highly damaging hydroxyl radicals. PCP (40 mg/kg bw) conjugates with cysteine residues of hemoglobin (Hb) and albumin to form semiquinone adducts which lower the oxygen-carrying ability of RBC and also disturb the osmotic balance in rat blood [65]. Chronic PCP toxicity lowers RBC count and hemoglobin level leading to anemia [21].

Several parameters of oxidative stress, including oxidation of proteins, lipids, and thiols, were determined to see if PCP induces oxidative stress in blood. GSH and GSH-dependent enzymes are essentially required for many reductive reactions in the body and regulate the concentration of free radicals/ROS. PCP interacts with GSH with the sequential replacement of chlorine molecules and also oxidizes its sulfhydryl group. TCBQ (tetrachlorobenzoquinone) can deplete up to four GSH molecules per TCBQ by interacting with multiple thiols. PCP metabolite TCHQ (tetrachlorohydroquinone) and also forms conjugates with GSH through non-enzymatic reactions that occur via direct chemical interaction [64]. GSH depletion could diminish the protective ability of cells against ROS, which interact with membrane proteins and lipids to alter their structure and properties [66]. LPO is the oxidation of polyunsaturated fatty acids present in membranes phospholipids and some of its products like malondialdehyde are themselves highly reactive and oxidize other cellular components and the extracellular matrix. Increased lipid peroxidation results in loss of membrane integrity, fluidity, and permeability and also alters activity of bound enzymes [67,68]. Under oxidative stress condition the endogenous hydroperoxides produced upon LPO may accelerate PCP metabolism to increase its toxicity and carcinogenicity [69]. Direct oxidation of side chain amino acids in polypeptide chains or adducts formed with LPO products generate carbonyl groups in proteins. Protein carbonylation irreversibly modifies the proteins to non-functional form and is used as a biomarker of protein oxidation [70]. Auto-oxidation and/or enzymatic oxidation of PCP in the liver of rats produces TCHQ and TCBQ followed by redox cycling that generates enormous amounts of H_2_O_2_ and hydroxyl radicals [71]. Oral administration of PCP increased H_2_O_2_ and NO production, NO can further react with O_2_^•-^ to produce peroxynitrite [72]. This enhanced level of ROS and reactive nitrogen species (RNS) likely results in oxidation of cellular components.

Mammalian cells are well equipped with non-enzymatic AOs that work by interrupting free radical chain reactions. Non-enzymatic AOs protect cells from oxidative damage by donating an electron or hydrogen atom to reduce metal ions and quench free radicals, respectively. The antioxidant power was greatly reduced in treated rats, which could be due to reduced levels of non-enzymatic AOs like GSH.

AO enzymes are responsible for the metabolism and/or stabilization of ROS and for maintaining the redox equilibrium in cells. AO enzymes protect aerobic organisms from oxidative damage and related disorders. SOD dismutates superoxide radicals into O_2_ and non-radical H_2_O_2_ while catalase further degrades this H_2_O_2_ to water and O_2_. Inhibition of SOD, catalase and GPx leads to increased levels of H_2_O_2_, hydroxyl radicals, peroxides and superoxides in blood [73,74]. GPx also decomposes H_2_O_2_ by using GSH as a reducing equivalent. The inhibition of GPx is likely due to the low concentration of GSH, which is used as a substrate in the breakdown of H_2_O_2_ to water [75]. TR maintains disulfide bonds of intracellular proteins in a reduced state while GR reproduces GSH from GSSG utilizing NADPH as a reducing compound. GR and TR work synergistically to combat oxidant attacks. PCP inhibited GR and TR activity dose-dependently. Thus, the activities of all major AO enzymes were significantly lowered in plasma and RBC of PCP-treated rats, when compared to control. ROS directly inhibit the active site of these enzymes or can react with transition metals in the prosthetic groups. This inhibition of AO enzymes compromises the ability of cells to protect themselves from oxidants because of lowered ROS scavenging capacity and consequent increase in ROS. The reduction in the level of GSH, a major non-enzymatic AO, will lead to enhanced formation of ROS and induction of oxidative stress condition. This will alter the functional and structural status of the cell and its membrane.

GST catalyzes the conjugation of GSH to xenobiotic substrates for detoxification. Increased GST could represent an adaptive response of the cell to combat enhanced ROS condition [74]. XO oxidizes hypoxanthine to xanthine and further into uric acid in the purine salvage pathways. XO is also a pro-oxidant enzyme involved in ROS generation. Increased XO activity was seen in plasma of PCP-treated rats. This, coupled with the inhibition of AO enzymes, will exacerbate the oxidative stress condition. Increased activity of plasma XO is associated with several pathologies [76]. PCP is known to cause depletion of AO enzymes which causes increased ROS and RNS levels in liver, kidneys, brain and RBC [77,78]. PCP-mediated ROS causes neuronal damage to hippocampus and degenerates retinal cells [79].

Oxidative damage modifies membrane lipids and proteins, alters membrane fluidity which affects the activities of bound enzymes. ATPases are integral membrane proteins that use energy from ATP hydrolysis to maintain essential *trans*-membrane action potential in virtually all living cells. They also maintain cellular integrity and function. PCP-induced ROS could have oxidatively modified the essential thiol groups of ATPases, resulting in decreased enzyme activity. PCP uncouples the oxidative phosphorylation in mitochondria isolated from rat liver, kidney and brain at low concentration (10 μM), and inhibits the ATPases at high concentrations (100 μM). PCP enhances the release of inorganic phosphate from ATP, which leads to ATP depletion [18,61]. PCP also restricted the uptake of Pi associated with the oxidation of α-ketoglutarate by rat liver mitochondria [80]. A significant amount of PCP binds to the cell membrane that induces the formation of both H_2_O_2_ and superoxide anion at a very high rate leading to oxidative damage. AChE is a significant marker of RBC membrane damage and its inhibition by PCP indicates the generation of oxidative stress [81].

Oxidative stress provokes metabolic failure which compromises cell viability by inhibiting the enzymes of glycolytic pathway and lowering ATP level [82]. PCP treatment significantly and dose-dependently inhibited the key enzymes involved in energy production and adversely affected cellular metabolism in rats. HK is the first enzyme of glucose metabolism that phosphorylates glucose to glucose 6-phosphatase. Oxidative stress condition leads to inactivation of HK which deprives cells of ATP. PK dephosphorylates phosphoenolpyruvate to pyruvate, yielding one molecule of ATP in the final step of glycolysis. ROS directly target the cysteine residue in PK active site [83]; hence the enzyme activity was reduced in PCP-treated rats.

G6PD catalyzes the first committed and rate-limiting step of phosphogluconate pathway by recycling the NADP^+^ to NADPH, a major cellular reductant. AO enzymes like TR and GR depend on NADPH which they use as substrate to catalyze their reactions and reduce ROS-mediated oxidative damage. Oral administration of PCP inhibited G6PD activity in RBC in PCP dose-dependent manner. This could be due to the inhibition of HK, whose product glucose 6-phosphate serves as a substrate for G6PD. Low concentration of glucose 6-phosphate will decrease G6PD activity and also the rate of hexose monophosphate shunt. Lowered G6PD activity will further reduce the NADPH level which will compromise the cellular reducing power leading to acute hemolytic anemia [84]. LDH reversibly converts lactate to pyruvate along with the reduction of NAD⁺ to NADH. Oxidative damage to the cellular membrane detaches the bound LDH and increases its activity. ACP hydrolyses organic phosphates in RBC and its activity was decreased in PCP dose-dependent manner.GLO-1 carries out the detoxification of reactive ketoaldehyde methylglyoxal and other reactive aldehydes that are produced as a normal part of metabolism. Increased level of endogenous methylglyoxal generates reactive carbonyl species (RCS) leading to toxicity and cellular damage. The level of RCS like methylglyoxal increases under oxidative stress condition and enhanced GLO-1 activity may be an adaptive response to quench these species [85].

Glucose is the primary and instant source of energy in human body that acts as starting substrate for many vital processes such as glycolysis, hexose-monophosphate shunt, etc. The increased glucose level in plasma from 100 mg/dl to 177 mg/dl could be due to inhibition, and less glucose degradation, of glycolysis in PCP-treated rats [86]. Inactivation of ATPases leads to reduced glucose co-transport within cells, this may also result in increased plasma glucose concentration. Inorganic phosphate (Pi) is required for normal cellular function, mineralization of bones, nerve function and muscle contraction. Lower Pi level in PCP-treated rats compared to control, will affect the electrolyte values and normal functions of the cell.

AST is a key enzyme in amino acid metabolism as it reversibly transfers an α-amino group between aspartate and glutamate. AST is a hepatic enzyme that is released in blood upon liver damage [66]. ALT is an important intermediate in cellular energy production that transfers an amino group from l-alanine to α-ketoglutarate. High ALT level in blood indicates liver disease/damage [17,73]. Increased ALT activity is due to damage to hepatic structure. The significant increase in AST and ALT levels in blood is a strong indicator of PCP-induced liver dysfunction or damage [66].

The significant increase in the activities of serum AST and ALT in PCP-treated rats suggested liver damage. This was confirmed by histopathological analysis of liver from control and PCP-treated rats. The hepatocytes in the liver of untreated control rats had normal structure, unclogged sinusoids and hepatocytes were lacking vacuoles [87]. The PCP-treated groups showed gradual changes in terms of distorted boundaries of hepatocytes, congestion of sinusoids and continuous increase in the number and size of intracellular vesicles and vacuoles. Fleische et al. [88] did a microscopic examination of the liver of PCP-treated rats and reported sinusoidal dilatation and an increase in extrahepatocytic space. Numerous intracytoplasmic vesicles, present in clusters can be seen to affect majority of the cells in the liver of rats treated with 150 mg PCP/kg bw. Darkening of nuclei in the PCP-treated groups can be related to the findings of Fleische et al. [88] that PCP enhanced the volume density and decreased the numerical density of nuclei in hepatocytes. This may be due to increased heterochromatin to euchromatin relation by PCP. Umemura et al. [89] reported an increase in cell proliferation and 8-oxodG level in mice liver, suggesting that PCP exposure increases ROS level that causes hepatic damage.

The strength of the study is that it is based on the evaluation of PCP hemotoxicity in rats, blood and its components are the primary markers of toxicity caused by xenobiotics entering the body. The assessment of the toxic effects of xenobiotics is more accurate in an animal model because it involves the internal environment of a living being, the findings of in vivo studies are considered more reliable or relevant. This study may also relate to hemotoxicity caused by other organochlorines or polychlorophenols. The limitation of this study is that the results should be correlated by analyzing the blood of persons who are occupationally exposed to PCP.

**Fig. 7:**
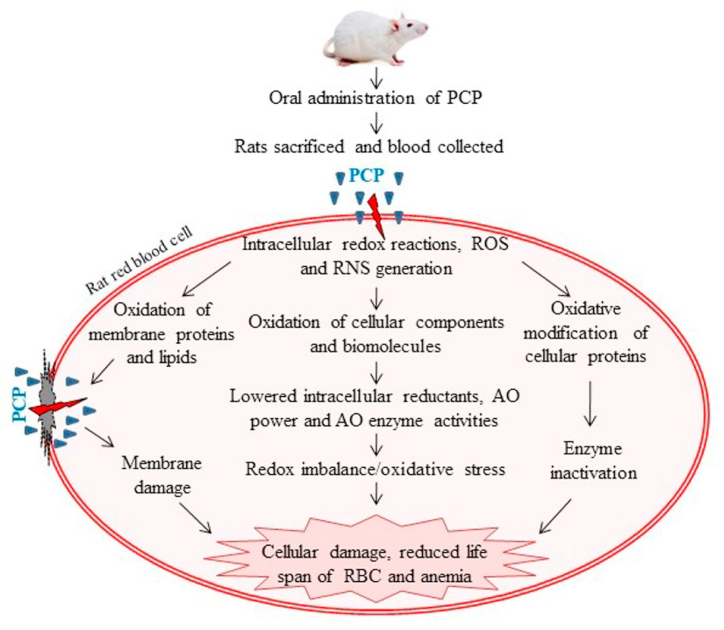
Schematic depiction of PCP-induced oxidative stress and hemotoxicity in rat blood. Oral administration of PCP to rats causes increased ROS and RNS formation, including H2O2, NO and hydroxyl radicals. This increase in reactive species causes oxidation of membrane lipids and proteins, oxidative modification of cellular proteins resulting in membrane damage and enzyme inactivation. Oxidative damage to membrane further increases the PCP influx in cells. This leads to generation of free radicals and ROS, reduces the cellular AOs and impairs the AO defense system. The diminished AO power results in a decreased ability of RBC to neutralize free radicals and ROS. This will lead to increased oxidative damage of cellular components, reduced lifespan of RBC and anemia. AO, antioxidant; PCP, pentachlorophenol; RBC, red blood cells; H2O2, hydrogen peroxide; ROS, reactive oxygen species; RNS, reactive nitrogen species. (For interpretation of the references to color in this figure legend, the reader is referred to the Web version of this article.)

In summary, oral administration of PCP enhances MetHb levels and increases the oxidation of thiols, proteins and lipids in rat blood due to generation of oxidative stress in the blood. It lowers the ability of cells to quench free radicals and reduces metal ions and inhibits the primary AO enzymes. Membrane damage is indicated by inhibition of bound enzymes while enzymes and pathways of glucose catabolism are also inhibited. PCP also causes hepatic damage as shown by increase in the level of its markers in plasma and histopathological analysis of liver. These results will help in elucidating the biochemical mode of toxic action of PCP so that appropriate methods can be designed to reduce its harmful effects (Fig. 7).

## 5 Summary

This study provides strong evidence of hemotoxicity and oxidative stress induced by PCP in blood of rats. PCP administration decreases AO activities, AO power, increases H_2_O_2_ and NO levels and oxidizes membrane proteins and lipids. It also leads to inhibition of enzymes in glucose metabolism. Thus, PCP significantly and dose-dependently induces oxidative damage in rat blood. PCP treatment also increases plasma AST and ALT levels and causes histological abrasions in liver indicative of liver damage.

No previous study has reported PCP-induced hemotoxicity in rats. PCP causes oxidative damage to RBC and plasma leading to anemia and other cardiovascular diseases. By understanding the molecular mode of PCP toxicity, we can devise methods to alleviate the harmful effects of PCP and other chlorophenols like using antioxidants as protective agents.

## Animal ethical clearance

This study was approved by the IAEC of the Department of Biochemistry, Faculty of Life Sciences, AMU and CPCSEA (Registration No. 714/GO/Re/S/02/CPCSEA).

## Author contribution statement

Nikhil Maheshwari: Conceived and designed the experiments; Performed the experiments; Analyzed and interpreted the data; Contributed reagents, materials, analysis tools or data; Wrote the paper.

Aijaz Ahmed Khan: Conceived and designed the experiments; Performed the experiments; Analyzed and interpreted the data.

Riaz Mahmood: Conceived and designed the experiments; Analyzed and interpreted the data; Wrote the paper.

Samreen Salam: Conceived and designed the experiments; Contributed reagents, materials, analysis tools or data.

## Funding statement

Nikhil Maheshwari was supported by Senior Research Fellowship from Council of Scientific and Industrial Research, New Delhi, {09/112 (0624)2K19-EMR-I}.

## Data availability statement

Data included in article/supplementary material/referenced in article.

## Declaration of competing interest

The authors declare the following financial interests/personal relationships which may be considered as potential competing interests: Nikhil Maheshwari reports was provided by Aligarh Muslim University. Nikhil Maheshwari reports a relationship with Aligarh Muslim University that includes: employment. All the authors declare no conflict of interest.
